# VE-MLM: A variable endmember-based multilinear mixing framework for crop FAPAR estimation using UAV multispectral imagery

**DOI:** 10.1016/j.plaphe.2026.100202

**Published:** 2026-04-04

**Authors:** Ningge Yuan, Yadong Liu, Chaoran Zhang, Yuanjin Li, Longfei Ma, Yi Peng, Xianting Wu, Renshan Zhu, Yan Gong, Shenghui Fang

**Affiliations:** a School of Remote Sensing and Information Engineering, Wuhan University, Wuhan, 430079, China; b Department of Geography and Resource Management, The Chinese University of Hong Kong, Hong Kong SAR, 999077, China; c South China Sea Sea Area and Island Center, Ministry of Natural Resources (South China Sea Standard Measurement and Information Center, Ministry of Natural Resources), Guangzhou, 510300, China; d Lab of Remote Sensing for Precision Phenomics of Hybrid Rice, Wuhan University, Wuhan, 430079, China; e College of Life Sciences, Wuhan University, Wuhan, 430079, China

**Keywords:** Spectral mixture analysis (SMA), Variable endmember (VE), Multilinear mixing model (MLM), FAPAR, Crops

## Abstract

The fraction of absorbed photosynthetically active radiation (FAPAR) is critical for characterizing crop photosynthetic capacity and growth status. Remote sensing technology based on unmanned aerial vehicles (UAVs) enables efficient estimation of FAPAR, but multiple scattering and transmission in the complex and dynamically changing crop canopy and background limit the accuracy of vegetation index (VI)-based methods. This study proposed an adaptive spectral unmixing framework VE-MLM for the multi-layer mixed scenarios, comprising three modules: (1) ***Variable Endmember Extraction***, building a spectral library of foreground (crop) and background endmembers, by extracting pure pixels on the R-NIR feature space and reducing redundancy using k-means and iterative endmember selection algorithm; (2) ***Iterative Unmixing***, iterating over foreground-background endmember combinations as input of the multilinear mixing model (MLM) pixel by pixel; (3) ***Optimal Selection***, selecting the optimal combination according to RMSE and outputting corresponding canopy abundance *A*_*f*_. Taking sorghum and rice as study objects, this study collected UAV multispectral images and field-measured FAPAR at multiple periods to validate the advantages of VE-MLM. The results demonstrated that compared to fixed-endmembers and linear/bilinear mixing models, VE-MLM always achieved excellent unmixing performance, effectively quantifying canopy contributions. The derived *A*_*f*_ mitigated the saturation and background interference that commonly existed in VI-based regression models and exhibited a higher correlation with FAPAR (sorghum: R^2^ = 0.900, rRMSE = 7.753%; rice: R^2^ = 0.807, rRMSE = 2.200%). In conclusion, VE-MLM has a great potential to address spectral variability, dynamic changes, and scene complexity in crop growth scenarios, providing a more accurate and generalizable approach for sorghum and rice FAPAR estimation in precision agriculture.

## 1 Introduction

The fraction of absorbed photosynthetically active radiation (FAPAR) is a key parameter that describes the proportion of solar radiation absorbed by vegetation during the process of canopy radiation transfer [1], which directly reflects the ability of vegetation canopy to capture and absorb light energy, and has been a crucial indicator for crop growth monitoring and yield prediction [2,3]. Therefore, accurate estimation and monitoring of FAPAR are essential for selecting and breeding high-light-efficient crop varieties, guiding crop cultivation activities, and coping with climate changes [4,5]. With the increasing demand for cost-effective and convenient monitoring methods, remote sensing (RS) has become an important technology to estimate FAPAR [6,7].

**Fig. 1:**
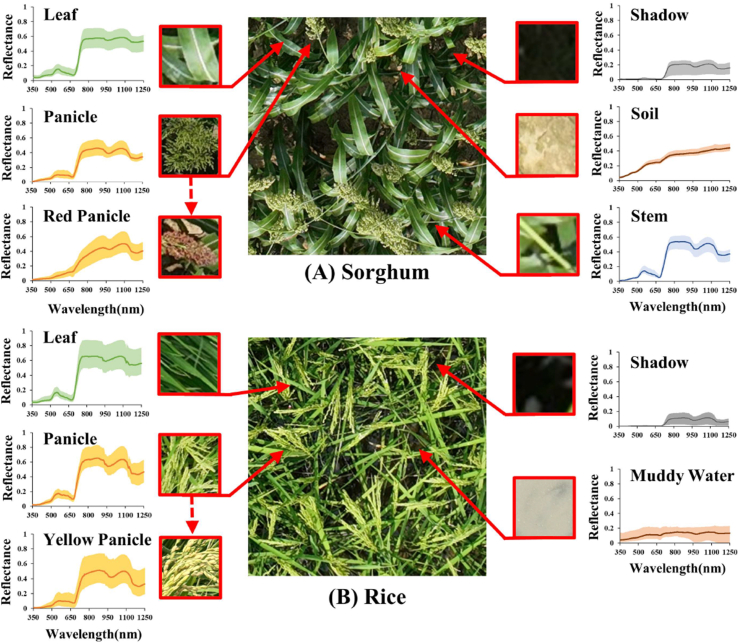
The field images and typical object spectra of (A) sorghum and (B) rice. The colored bars are the spectral variation ranges. Note: The in situ spectra were measured from the crop fields using an ASD Field Spec 4 spectrometer (Analytical Spectral Devices Inc., Boulder, CO, United States).

At present, there are two primary ways to derive FAPAR from RS data, including physical and empirical methods. Physical methods use gap probability [8], radiative transfer equations [9,10], or recollisional probability [11,12] to describe the light transfer process and model the relations between FAPAR and canopy reflectance. But the physical models are usually complex and require many input parameters, which limits their practical application [8,13]. Empirical methods derive linear or nonlinear relations between simulated or field-measured FAPAR and the vegetation index (VI), such as normalized difference vegetation index (NDVI) [14], green normalized difference vegetation index (GNDVI) [15], enhanced vegetation index (EVI) [16], and so on. Empirically derived relationships are simple and efficient in application. However, the signals received by sensors are usually the result of multiple scattering and reflection of light between the canopy and irrelevant backgrounds, such as soil. Consequently, the imagery is dominated by mixed pixels, which contain spectral contributions from multiple components [17]. Additionally, plant structures change dynamically during growth stages (Fig. 1). These factors affect the establishment of empirical relationships, leading to limited universality and generalizability of VI-based approaches [18,19]. Spectral mixture analysis (SMA) provides a new approach to overcoming the limitations of VIs.

SMA can separate signals of different ground objects from pixel spectra and obtain the weighting of each object or endmember, namely abundance, by inputting spectra of endmembers into the spectral mixture model and solving the model [20]. Traditional SMA uses a fixed set of endmembers and linear mixing model (LMM), and works reasonably well in areas with relatively homogeneous land cover [21,22]. To address complex environments, the applicability of SMA in the inversion of crop parameters, such as FAPAR, faces two main challenges. On the one hand, spectral variability is a common phenomenon in RS images. The same material could have different spectral responses in a scene across space and time, referred to as spectral variability, which leads to estimation error of the abundance in SMA [17,23]. To minimize its effect, the multiple endmember spectral mixture analysis (MESMA) strategy was specifically conceived by selecting and utilizing distinct spectra for each endmember class [24], one of the most widely used and successful methods [25,26]. In MESMA, the primary source of endmembers is the spectral library, whose building relies on field measurement or manual extraction [27]. However, pure endmembers extracted from the same observed image have been proved to be optimal [27,28]. Typically, image-based automatic endmember extraction algorithms either assume the existence of pure pixels, which are defined as those occupied by a single homogeneous land cover class and generally scarce in real imagery [29], or generate virtual endmembers, which may compromise the quantitative analysis accuracy of abundance [30]. Additionally, these endmember extraction methods are generally unable to obtain all representative endmembers when spectral variability exists, which may lead to an unmixing error [31]. On the other hand, photons undergo multiple scattering and transmission in fields, especially in vegetation and mineral mixtures [32,33]. Considering the light propagation path and the interactions between endmembers, spectral mixing models establish mathematical relationships with their own restrictions and underlying physical reasoning among pixel spectra, endmembers, and abundances. The LMM assumes that light interacts with only a single material, where abundance represents the area of the material, and is suitable for simple fields [34]. However, in a multi-layer hybrid scene such as farmland and urban areas, incoming light ray undergoes multiple interactions within the scene, limiting the application accuracy of LMM. Consequently, nonlinear mixing models have emerged and considered quadratic or multiple interactions, including the bilinear mixing model (BMM) [[35], [36], [37]], the high-order linear mixing model [38], and so on. Due to the nonlinear terms, the physical meaning of abundance is not the area of the material in two-dimensional space but relatively ambiguous. Among these nonlinear models, the multilinear mixing model (MLM) introduces the photon recollisional probability and describes the light path with a clear underlying physical reasoning [32,38]. The ultimate abundance derived from MLM represents the probability of light interacting with the material, considering multiple scattering and transmission.

SMA is beneficial for improving the classification accuracy of ground objects [39], and enhancing the ability to identify small targets such as crop organs [40]. Currently, it has been widely applied in large-scale mapping such as land classification [41], fire severity assessment [42], as well as specific application fields like shadow removal [43] and leaf disease detection [44]. For the application of SMA in crop growth fields, some studies have shown that the abundance is strongly correlated with the proportion of incident solar radiation intercepted by plant canopy [45,46]. However, further exploration is needed regarding model accuracy [47] and the physical implications of abundance [46,48].

As representatives of C4 and C3 plants, respectively, sorghum and rice exhibit distinct canopy structures, photosynthetic pathways, and growth environments [49,50]. After emergence, leaves and stems continuously interlace in various postures in three-dimensional space. With the growth, the canopy volume expands until the panicles emerge from the top. The sorghum canopy is relatively sparse with larger panicles at the top of plants; meanwhile, the rice canopy is sparse in the early stage until the late jointing stage, when it becomes dense and causes row closure [51]. And during the mature stage, the panicles of sorghum and rice turn red and yellow, respectively. Soil or muddy water (background), together with crop canopies (foreground), form dynamically changing multi-layered mixed scenes (Fig. 1). Inevitably, the complexity and dynamic variability of such scenes can affect the establishment of empirical relationships between VI and FAPAR [52,53]. Accurately describing the scattering and transmission process and separating the foreground and background according to the actual crop growth status are critical challenges that must be tackled in retrieving FAPAR from reflection spectra.

To address the problems described above, it is necessary to develop an adaptive unmixing method suitable for crop fields that can better account for endmember variability and more accurately simulate light propagation throughout the complex scene. Consequently, this study developed an adaptive unmixing framework, VE-MLM, for accurate crop FAPAR estimation. The main contributions of this study are as follows.

• An adaptive variable endmember strategy is proposed to accommodate the dynamic changes of crop canopies. By constructing a representative spectral library, the framework effectively addresses intra-class spectral variability, enabling the model to adaptively match the diverse spectral features across different growth stages.
• The complex optical transmission process is rigorously simulated via the MLM model. The framework explicitly accounts for the multiple scattering and transmission interactions between the canopy and background, providing a more physically rigorous description than commonly used linear models.
• The accuracy and robustness of FAPAR estimation are significantly improved. The derived canopy abundance (*A*_*f*_) effectively mitigates the saturation effects and background interference inherent in VIs, demonstrating superior performance over both empirical VIs and machine learning (ML) models in multi-period sorghum and rice experiments.

## 2 Materials and methods

### 2.1 Study area

**Fig. 2:**
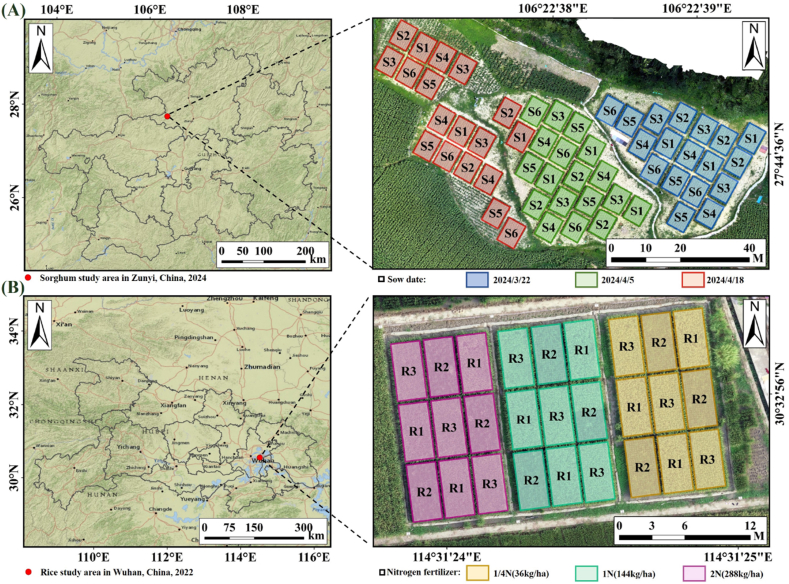
Overview of the (A) sorghum and (B) rice study area. Note: S1, S2, S3, S4, S5 and S6 represent six sorghum varieties; R1, R2 and R3 represent three rice varieties; and the rectangles with the different colors represent different sowing dates or nitrogen treatment levels for sorghum or rice.

This study conducted multiple field experiments with sorghum and rice as research objects, respectively (Fig. 2). The sorghum study area was situated in Zunyi City, Guizhou Province, China (27°42′N, 106°22′E). The region has a central subtropical humid monsoon climate, with perennial drought, changeable weather, and an average altitude of 880 m. It is one of the main producing areas of glutinous sorghum in China. In 2024, 54 plots were designed in the experimental area, each with a width of 5.4 m and a length of 7.2 m, and the planting density was 11.1 plants/m^2^. The experiment set up 3 sowing date levels, and each level planted 6 representative glutinous sorghum varieties such as Hongyingzi and Hongyingzi 519, with 3 replicates. The sorghums were sown on 2024/3/22, 2024/4/5, and 2024/4/18 respectively. According to local standards, the same field management plan, including irrigation, fertilization, and pest control, was executed for each plot.

The rice study area was located in Wuhan City, Hubei Province, China (30°33′ N, 114°32′ E). The region has a subtropical monsoon climate with abundant heat and an average altitude of 39 m, where rice is widely cultivated. In 2022, 27 plots were planned in the experimental area, each with a width of 2.8 m and a length of 5.2 m, and the planting density was 28 plants/m^2^. The experiment set up 3 nitrogen fertilizer levels (1/4N: 36 kg/ha, 1N: 144 kg/ha, 2N: 288 kg/ha). Each level included two indica rice varieties (Fengliangyou 4 and Luoyou 9348) and one japonica rice variety (Changjingyou 582), with 3 replicates. All rice seedlings were transplanted on June 14, 2022. Except for the fertilizer application, the same field management was implemented for each plot.

### 2.2 Data collection

#### 2.2.1 Multispectral UAV images and pre-processing

In the two experiments, multispectral images were acquired by a DJI M600 PRO hexacopter (DJI, Shenzhen, Guangdong, China) (Fig. S1A) equipped with a Micro-MCA 12 multispectral sensor (Tetracam Inc., Chatsworth, CA, USA) (Fig. S1B). The sensor consists of 12 independent custom lenses covering vegetation-sensitive wavelength bands, specifically 490, 520, 550, 570, 670, 680, 700, 720, 800, 850, 900 and 950 nm. Among these, the first 10 bands have a bandwidth of 10 nm, the 900 nm band has a bandwidth of 20 nm, and the 950 nm band has a bandwidth of 40 nm. Each flight was arranged between 10:00 a.m. and 2:00 p.m. local time, when illumination was stable and the changes in the solar zenith angle were minimal.

In 2024, a total of 6 flights were conducted on 6/6, 6/15, 6/26, 7/7, 7/16 and 7/25. In 2022, 5 flights were carried out on 7/11, 7/22, 8/2, 8/15 and 8/25. The data from the two experiments both covered the jointing, booting and heading stages. Due to local climatic constraints, all images were collected under clear, windless, and cloudless conditions, except for 2024/6/6 and 2024/6/26, which were cloudy. In addition, we collected imagery data in the crop maturity stage to further discuss the applicable scenarios of our method, on 2024/8/6, 2024/8/14 and 2022/9/5.

During UAV flights, eight approximately Lambertian standard reflectance panels were laid in the experimental field, with reflectance values of 0.03, 0.06, 0.12, 0.24, 0.36, 0.48, 0.56, and 0.80, respectively (Fig. S1E). To ensure one single UAV image could cover all crop plots and calibration panels in the experimental area, the UAV flight altitude was set to 200 m with a spatial resolution of 10.96 cm in 2024 and 110 m with a spatial resolution of 6.03 cm in 2022 (Fig. S1D). After acquiring full-coverage images, close-range multispectral images with a spatial resolution of 1.09 cm (Fig. S1C) were immediately captured at an altitude of about 20 m, covering crop canopy and all calibration panels to obtain organ-level pure pixels.

By referring to standard panels, the piecewise linear correction method was applied to transform digital numbers (DNs) to surface reflectance (ρ_λ_) (Eq. (1)). Specifically, regions of interest (ROIs) were delineated on each calibration panel to obtain average DN values, which were then substituted into Eq. (1) along with standard reflectance values. The gain coefficients (Gain_λ_) and offset coefficients (Offset_λ_) at the wavelength *λ* were calculated by applying the least-squares method and used in radiometric calibration.

(1) $$\rho_{\lambda }=\left\{ \begin{array}{c} {Gain}_{\lambda }\times {\;\text{DN}}_{\lambda },\rho_{\lambda }\leq 0.03\\ {Gain}_{\lambda }\times {\;\text{DN}}_{\lambda }+{\text{Offset}}_{\lambda },\rho_{\lambda }>0.03 \end{array}\right.$$

For each plot, we respectively defined rectangles with the same size as ROIs in the UAV image after radiometric calibration, using the ENVI 5.6 software (NV5 Geospatial, Broomfield, Colorado, USA). The plot-level VI was calculated by averaging all the per-pixel reflectance values within the ROI. A total of 15 VIs commonly used to estimate FAPAR or monitor vegetation status were selected, as shown in Table S1. Furthermore, these 15 VIs served as input features for three representative ML models-random forest (RF), support vector regression (SVR), and artificial neural networks (ANN)-to perform non-linear FAPAR estimation. To ensure robust evaluation, a 5-fold cross-validation strategy was applied.

#### 2.2.2 Field FAPAR data

In this study, LI-190R quantum sensor and LI-191R line quantum sensor (LI-COR Inc., Lincoln, NE, USA) were used to measure the FAPAR data of each plot (Fig. S1F). The LI-190R sensor was horizontally placed 50 cm above the canopy top to receive incident PAR (PAR_in_) and reflected PAR from the top of the canopy (PAR_out_) (Fig. S1G). The LI-191R sensor was horizontally placed 10 cm above the ground to sense light within a 1-m range, capturing canopy-transmitted PAR (PAR_ct_) received at the canopy bottom and soil-reflected PAR (PAR_sr_) (Fig. S1G). Three sets of data were collected for each plot, with the LI-191R sensor oriented parallel to the row direction (0°), diagonally to the row direction (45° and −45°), respectively (Fig. S1H). The three sets of data were respectively substituted into Eq. (2) to calculate the FAPAR, and the average of the three results was the final FAPAR of the plot.

(2) $$FAPAR=\frac{PAR_{in}-PAR_{rc}-PAR_{ct}+PAR_{sr}}{PAR_{in}}$$

On the day of the UAV flight, FAPAR data were collected from field plots between 9:30 and 10:30 local time. Due to experimental constraints, FAPAR was collected across different sowing dates and varieties, resulting in a total of 281 samples for the 2024 sorghum experiment. For the 2022 rice experiment, FAPAR was collected across all plots, resulting in a total of 162 samples.

### 2.3 VE-MLM framework

**Fig. 3:**
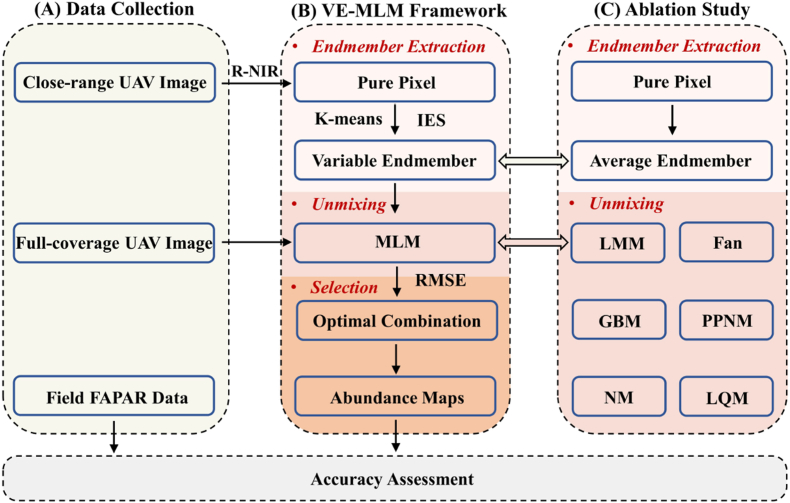
The workflow diagram of this study including the proposed VE-MLM framework and the ablation study. Note: IES, iterative endmember selection algorithm; MLM, multilinear mixing model; LMM, linear mixing model; Fan, fan model; GBM, generalized bilinear model; PPNM, polynomial post-nonlinear model; NM, Nascimento model; LQM, linear quadratic model.

To address the dynamic changes and complexity of crop growth fields, the VE-MLM framework was designed to obtain crop abundance (Fig. 3), which specifically includes three modules: (1) **Variable Endmember Extraction**, adaptively establishing an endmember spectral library based on close-range UAV images, containing *m* foreground spectra and *n* background spectra; (2) **Iterative Unmixing**, iterating through all potential combinations (*m* × *n*) of the spectral library and using the MLM model to unmix the full-coverage images; (3) **Optimal Selection**, selecting the optimal endmember combination according to root mean square error (RMSE) and outputting the corresponding abundance.

In addition, to verify the necessity of key modules in VE-MLM, an ablation study was conducted (Fig. 3). For the Variable Endmember Extraction module, one foreground endmember (FE) and one background endmember (BE) were obtained by averaging pure pixels, serving as a fixed set of endmembers for subsequent unmixing. For the Iterative Unmixing module, one linear mixing model and five typical bilinear mixing models were used as contrastive models. Finally, a comparative analysis was performed on the unmixing errors and the correlation between FAPAR and foreground abundances derived from different unmixing strategies.

#### 2.3.1 Definition of the variable spectral library

**Fig. 4:**
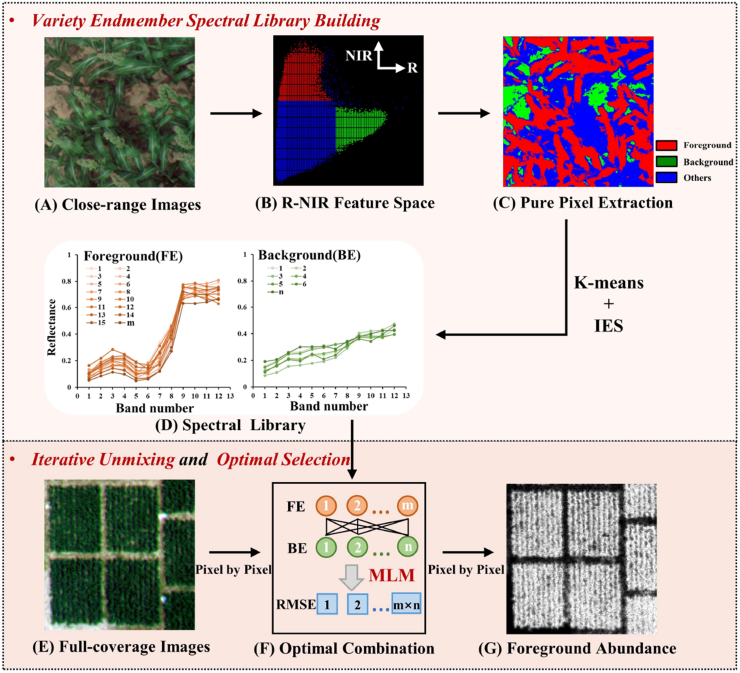
Flowchart of the proposed VE-MLM framework.

In sorghum and rice growth fields, the foreground primarily consists of the green organs of crops (leaves, stems and panicles), which exhibit significant vegetation spectral characteristics: low reflectance in the red (R) band and high reflectance in the near-infrared (NIR) band. The background consists of soil or muddy water, showing higher reflectance in the R band and lower reflectance in the NIR band than foreground spectra (Fig. 1). It is worth noting that while VIs (e.g., NDVI) are commonly used for vegetation extraction, our tests indicated that threshold segmentation methods based on VI struggled to effectively distinguish between pure sunlit leaves and shadowed components (shadowed leaves/soil/water), often resulting in the inclusion of impure pixels. In contrast, considering the differences in reflectance characteristics between the foreground and background, we constructed an R-NIR two-dimensional triangular feature space to extract pure pixels of the foreground and background contained in the close-range images (Fig. 4A). Typically, feature points representing pure pixels of foreground were located at the upper corner of the triangular space (red points in Fig. 4B), pure pixels of background at the right corner (green points in Fig. 4B), and mixed pixels at other positions within the feature space (blue points in Fig. 4B). The R-NIR feature space endmember extraction was operated using the n-dimensional visualization space tool in ENVI 5.6.

A large number of foreground and background spectra were extracted based on the R-NIR feature space (Fig. 4C). However, these pure pixel spectra exhibit two coexisting characteristics: intra-class repeatability and intra-class variability. The former refers to the high spectral similarity observed among the vast number of extracted pixels [26]. The latter refers to the spectral distinctions within the same class caused by factors such as illumination geometry, leaf angle, and soil moisture [17]. To address the computational burden caused by high repeatability while preserving the representative features of variability, the K-means algorithm was employed to cluster the endmembers. This step effectively compresses the raw data into representative centroids, thereby preliminarily reducing data redundancy and the number of pure pixels within the scene. Specifically, the numbers of foreground and background pure pixels were typically set to 1000 and 500, respectively. Subsequently, the iterative endmember selection (IES) algorithm [54] was applied to quickly screen out endmember spectra that best represent object features while capturing intra-class spectral variability, obtaining the optimal subset of the original spectral library [55]. Thus, for each image, an optimal variable endmember (VE) spectral library containing only two types of endmembers (FE and BE) was constructed based on close-range UAV images (Fig. 4D). Herein, the number of foreground endmembers was *m*, and background endmembers was *n*, where *m* > *n* and neither *m* nor *n* equaled 1 in our experiments. The IES algorithm was implemented using VIPER-Tool v2.1 within ENVI 5.6 [56].

#### 2.3.2 Unmixing with MLM

**Fig. 5:**
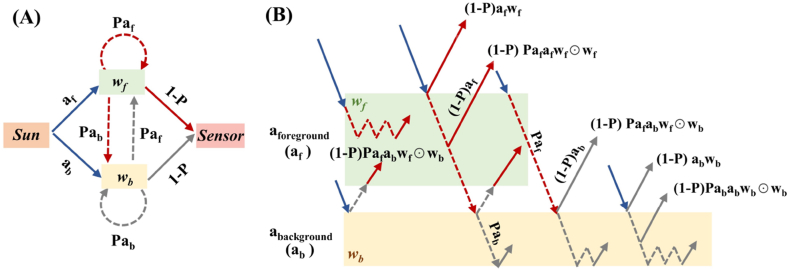
Schematic of MLM for the field scenario. Diagram describing (A) the transition probabilities between sun, sensor, foreground, and background; (B) multiple interactions of photons with foreground and background.

The MLM [32] describes multiple interactions of light within a scene as a discrete Markov process (Fig. 5). Assuming only two kinds of materials exist in crop growth fields: the foreground consists of plant organs such as panicles, leaves, and stems with abundance *a*_*f*_ and albedo *w*_*f*_; the background consists of soil or muddy water with abundance *a*_*b*_ and albedo *w*_*b*_. The MLM assumes that light interacts with at least one material after entering the scene, and the probability of interaction with the material is proportional to its abundance; after each interaction with a material, photons continue to reflect within the scene with probability *P* or escape the scene and enter the sensor with probability (1−*P*); and when a light ray is scattered by material *i*, its intensity varies with albedo *w*_*i*_ ∈ [0,1]^d^.

The single reflection signal received by the sensor, i.e., the first-order term of the model, can be expressed as,

(3) $$x_{1}=\left(1-P\right)a_{f}w_{f}+\left(1-P\right)a_{b}w_{b}$$

The secondary interaction signal received by the sensor, i.e., the second-order term of the model, can be expressed as,

(4) $$x_{2}=\left(1-P\right)Pa_{f}a_{f}w_{f}⊙w_{f}+\left(1-P\right)Pa_{f}a_{b}w_{f}⊙w_{b}+\left(1-P\right)P{a_{b}a}_{f}w_{b}⊙w_{f}+\left(1-P\right)Pa_{b}a_{b}w_{b}⊙w_{b}$$

where ⊙ is the Hadamard product of two vectors.

By analogy, the reflectance is obtained by the sum of contributions from all possible paths, so that the pixel reflectance *x* can be expressed as,

(5) $$x=x_{1}+x_{2}+x_{3}+\ldots =\left(1-P\right)\sum_{i=1}^{p}\;a_{i}w_{i}+\left(1-P\right)P\sum_{i=1}^{p}\;\sum_{j=1}^{p}\;a_{i}a_{j}w_{i}⊙w_{j}+\left(1-P\right)P^{2}\sum_{i=1}^{p}\;\sum_{j=1}^{p}\;\sum_{k=1}^{p}\;a_{i}a_{j}a_{k}w_{i}⊙w_{j}⊙w_{k}+\ldots =\left(1-P\right)y+\left(1-P\right)Py^{2}+\left(1-P\right)P^{2}y^{3}+\ldots =\left(1-P\right)y+Py⊙\left(\left(1-P\right)y+P\left(1-P\right)y^{2}+\ldots \right)=\left(1-P\right)y+Py⊙x$$

where $y=\sum_{i=1}^{p}\;a_{i}w_{i}$, *p* is the number of endmember classes. The solution is given by

(6) $$x=\frac{\left(1-P\right)y}{1-Py}=\frac{\left(1-P\right)\sum_{i=1}^{p}\;a_{i}w_{i}}{1-P\sum_{i=1}^{p}\;a_{i}w_{i}}$$

When there is only a single material *i* with albedo *w*_*i*_ in a pixel, then Eq. (6) reduces to

(7) $$x_{i}=\frac{\left(1-P_{i}\right)w_{i}}{1-P_{i}w_{i}}$$

where *x*_*i*_ is the pure pixel reflectance, which is also the endmember spectrum *e*_*i*_ of the material. Therefore, as long as the recollision probability *P*_*i*_ is obtained, the relationship between its endmember spectrum *e*_*i*_ and the corresponding albedo *w*_*i*_ can be determined as follows.

(8) $$w_{i}=\frac{e_{i}}{P_{i}e_{i}+1-P_{i}}$$

In most practical scenarios, it can be assumed that the nonlinear effect of the same type of ground object is relatively small, so for all *i*: *e*_*i*_ ≈ *w*_*i*_. However, for this study, in crop growth environments, the nonlinear effects between leaves are considered not to be neglected. Substituting Eq. (8) into Eq. (6) gives the relationship between the endmember *e*_*i*_ and the pixel reflectance *x*.

In our experiments, the input variables were the foreground endmember spectrum *e*_*f*_, the background endmember *e*_*b*_, and the pixel reflectance *x* to be unmixed. The MATLAB function FMINCON was used to perform optimization pixel by pixel. Besides *P* < 1, the abundances were subject to the abundance sum-to-one constraint (ASC) and the abundance nonnegativity constraint (ANC). Finally, the optimal solution for the foreground abundance *a*_*f*_, background abundance *a*_*b*_ and the recollision probability *P* of the mixed pixel were obtained and output.

#### 2.3.3 Optimal endmember combination selection

The endmember spectra from the spectral library established in Section 2.3.1 and the full-coverage images (Fig. S1D) were input into the MLM introduced in Section 2.3.2 using an exhaustive cross-pairing strategy (Fig. 4F). Specifically, the spectral library contains *m* foreground endmembers and *n* background endmembers. By iteratively inputting one foreground spectrum and one background spectrum into the MLM, a total of *m* × *n* endmember combinations are generated. Each endmember combination, together with the reflectance of pixel *x*, is input into the MLM for unmixing. For a given pixel *x*, this process yields *m* × *n* corresponding results. Subsequently, the RMSE is calculated one by one to evaluate the unmixing residual of each combination for each pixel as follows:

(9) $$RMSE=\sqrt{\frac{\sum_{i=1}^{N}{\left(x_{i}-{x\left(a,e,P\right)}_{i}\right)}^{2}}{N}}$$

where *x(a,e,P)* is the reconstructed spectrum calculated by the abundance *a*, endmember spectrum *e* and the recollision probability *P* into the MLM model (Eq. (6) and Eq. (8)); *N* is the number of spectral bands.

Ultimately, each pixel yields *m* × *n* RMSE values, which are obtained with a possible endmember combination. The endmember combination and corresponding abundances used in the final result are determined based on the minimum RMSE (Fig. 4G).

#### 2.3.4 Ablation study

To demonstrate the advantages and effectiveness of the VE-MLM framework in crop growth fields, the ablation study was designed for its two key modules.

For the **Variable Endmember Extraction** module, it was necessary to extract a set of fixed image endmembers for comparison with the VE spectral library. In this study, we calculated the average reflectance of pure pixels in different classes, which were extracted from the R-NIR space established in Section 2.3.1, and obtained one FE and one BE. These two AEs served as a fixed set of spectra directly fed into the mixing model for the corresponding full-coverage image. Owing to the uniqueness of the endmember combination, subsequent RMSE evaluation for optimal endmember selection was eliminated.

For the **Unmixing** module, the LMM and five BMMs were selected as comparative models, with their formulas listed in Table S2. The LMM only considers the single reflection of light with ground objects in the scene. The BMMs further consider secondary scattering and transmission of light, and different BMMs have distinct definitions of the bilinear term. Specifically, the Fan model, the generalized bilinear model (GBM) and the polynomial post-nonlinear model (PPNM) assume the probability of light interacting with any two endmembers has to be proportional to the weight of the linear terms in the scene, and GBM and PPNM introduce adjustment parameters for nonlinear terms in different ways. The Nascimento model (NM) and the linear-quadratic model (LQM) assume that the bilinear interaction terms can be considered as extra endmembers, with interaction probability directly unrelated to abundances. Additionally, Fan, GBM, and NM do not consider self-interactions. The models were solved by fully constrained least squares (FCLS) or subgradient-based algorithms (Gradient) recommended in the papers.

### 2.4 Accuracy assessment

In order to evaluate the performance of unmixing frameworks under the same criteria, this study focused on two aspects: the unmixing quality and the correlation between the foreground abundance and the field-measured FAPAR.

#### 2.4.1 Unmixing accuracy

The relative error (RE) and spectral angle distance (SAD) were used to evaluate the unmixing accuracy of the model. A higher RE or SAD indicates more information loss during unmixing. The RE and SAD are respectively defined by

(10) $$RE={\left\|x-x\left(a,e,P\right)\right\|}_{2}^{2}$$

(11) $$SAD=\arccos\left(\frac{x^{2}x\left(a,e,P\right)}{{\left\|x\right\|}_{2}{\left\|x\left(a,e,P\right)\right\|}_{2}}\right)$$

where *x* represents the original pixel spectrum, and *x(a,e,P)* denotes the reconstructed spectrum recalculated based on abundance *a*, the endmember spectrum *e* and the recollision probability *P*.

In addition, we calculated the standard deviation (Std) of the RE and SAD. The Std serves as a critical indicator of the model's stability and robustness across the temporal and spatial domains. A lower Std implies that the unmixing framework maintains consistent performance across different crop varieties and dynamic growth stages, without exhibiting significant fluctuations or extreme errors in complex scenarios. The specific calculations are as follows:

(12) $$Std=\sqrt{\frac{\sum_{i=1}^{S}{\left(x\left(a,e,P\right)-\bar{x\left(a,e,P\right)}\right)}^{2}}{S-1}}$$

where *S* denotes the number of samples.

Since the focus was more on the unmixing quality of the crop field, the model performance was assessed by calculating the mean and standard deviation of the plot-level RE and SAD, where each value represented the average of all pixels within the corresponding ROI.

#### 2.4.2 Data analysis between UAV data and FAPAR

The Pearson correlation coefficient was used to evaluate the correlation between the field-measured FAPAR data and foreground abundance data, which were obtained by different unmixing frameworks. A higher *r* value indicates a stronger correlation between abundance and FAPAR. The Pearson correlation coefficient can be expressed by

(13) $$r=\frac{\sum_{i=1}^{S}\left(X_{i}-\bar{X}\right)\left(Y_{i}-\bar{Y}\right)}{\sqrt{\sum_{i=1}^{S}{\left(X_{i}-\bar{X}\right)}^{2}\sum_{i=1}^{S}{\left(Y_{i}-\bar{Y}\right)}^{2}}}$$

where *X* represents abundance data or VI in our experiments, *Y* represents FAPAR data, and *S* denotes the number of samples.

We developed linear regression models between the foreground abundance or VIs and FAPAR across multiple growth periods. The coefficient of determination (R^2^) and relative root mean square error (rRMSE) were calculated for each FAPAR estimation model to assess the performance. The specific calculations are as follows:

(14) $$R^{2}=1-\frac{\sum_{i=1}^{s}{\left({\hat{Y}}_{i}-Y_{i}\right)}^{2}}{\sum_{i=1}^{s}{\left({\bar{Y}}_{i}-Y_{i}\right)}^{2}}$$

(15) $$\mathrm{rRMSE}=\frac{\sqrt{\frac{1}{S}\sum_{i=1}^{S}\;{\left(Y_{i}-{\hat{Y}}_{i}\right)}^{2}}}{\sum_{i=1}^{S}\;{Y_{i}}^{2}}\times 100$$

where *Y*_*i*_ is the true value, *Y*_*i*_ is the predicted value.

## 3 Results

### 3.1 Verification of unmixing accuracy

To evaluate the accuracy of the VE-MLM framework and verify the effectiveness of its key modules, this study made comparisons between the VE and AE, and between the MLM and six other linear or bilinear models. And we conducted comparative analyses of unmixing performance from both qualitative and quantitative perspectives. The more effective the unmixing framework, the greater its potential to accurately simulate the propagation of light in the scene.

**Fig. 6:**
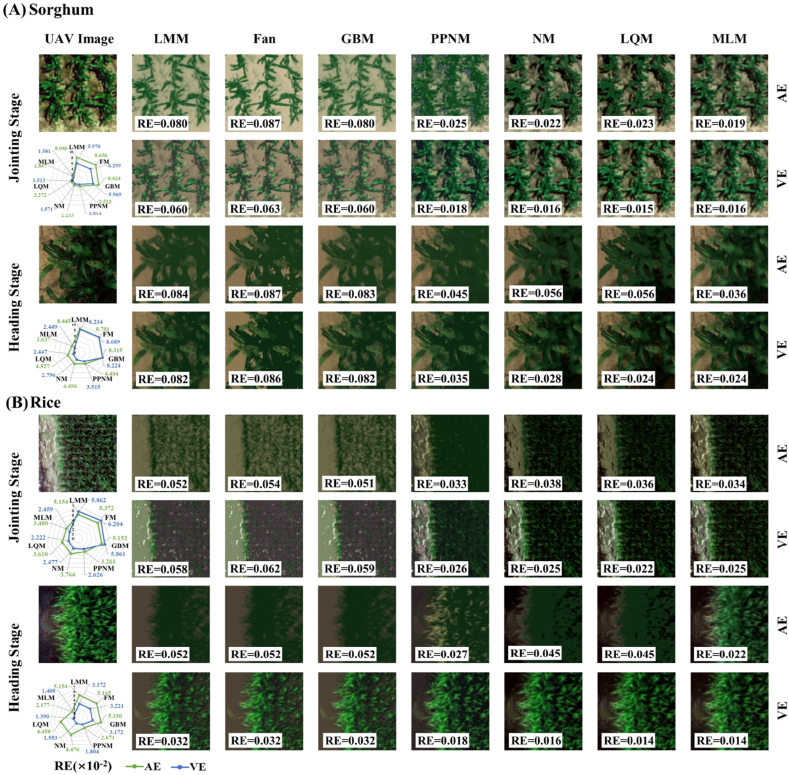
The original UAV images and reconstructed images of different SMA frameworks of (A) sorghum and (B) rice at the jointing stage and heading stage. Note: AE means fixed average endmembers; VE means the variable endmember spectral library; LMM, FM, GBM, PPNM, NM, LQM and MLM are different mixing models; RE means relative error.

For qualitative evaluation of the models, to intuitively assess unmixing performance, we reconstructed images *x(a,e,P)* according to the corresponding endmember and mixing model. A greater similarity between the reconstructed and original images indicates superior unmixing performance. Taking close-range images from the jointing and heading stages as examples, Fig. 6 displays reconstructed true color images of sorghum and rice, using different unmixing frameworks.

As shown in Fig. 6, compared with the original UAV images, VE-NM, VE-LQM, and VE-MLM achieved the highest fidelity for both sorghum and rice. VE-PPNM and AE-MLM also demonstrated strong unmixing performance. For all mixing models, VE visually outperformed AE in reconstructing crop growth scenes and capturing details of heterogeneous ground objects, such as leaf hotspots caused by illumination variations and soil differences resulting from the moisture content. Regarding model applicability, LMM, Fan, and GBM exhibited relatively poor performance, particularly for shadows, whereas NM, LQM, and MLM achieved significantly better results for main materials including leaves, panicles, shadows, and soil or water. Quantitatively, the RE metrics corroborated these visual observations. Consistent with the visual results, VE-NM, VE-LQM, and VE-MLM achieved the lowest mean RE values for both crops across the jointing and heading stages.

For further quantitative accuracy evaluation, the RE and SAD were calculated for full-coverage UAV images using Eq. (10) and Eq. (11). Table 1 lists the average values and standard deviations of multi-period images, which were calculated separately from all sorghum images and rice images. A lower value indicates better performance of the unmixing framework.

**Table 1:** RE and SAD for the sorghum and rice experiments (Mean ± Std).

	RE of Sorghum		RE of Rice		SAD of Sorghum		SAD of Rice	
	AE ( × 10^−2^)	VE( × 10^−2^)	AE( × 10^−2^)	VE( × 10^−2^)	AE( × 10^−2^)	VE( × 10^−2^)	AE( × 10^−2^)	VE( × 10^−2^)
LMM	9.271 ± 4.043	8.129 ± 2.814	4.681 ± 1.605	4.276 ± 0.800	20.148 ± 5.431	23.383 ± 5.573	17.721 ± 6.577	16.078 ± 4.545
FM	10.054 ± 4.467	8.770 ± 3.062	4.922 ± 1.591	4.633 ± 0.859	21.715 ± 8.555	23.044 ± 8.455	18.624 ± 6.651	17.369 ± 4.869
GBM	9.272 ± 4.043	8.129 ± 2.814	4.681 ± 1.605	4.276 ± 0.800	20.159 ± 5.432	23.406 ± 5.584	17.762 ± 6.578	16.133 ± 4.573
PPNM	3.222 ± 1.225	2.663 ± 0.931	3.866 ± 1.936	2.417 ± 0.591	15.866 ± 5.319	13.507 ± 5.116	14.821 ± 7.214	9.247 ± 2.401
NM	2.305 ± 0.513	1.491 ± 0.420	2.600 ± 0.991	1.540 ± 0.605	12.126 ± 5.452	7.7795 ± 3.770	9.677 ± 2.953	5.716 ± 1.989
LQM	2.172 ± 0.490	1.358 ± 0.394	2.043 ± 0.682	1.058 ± 0.394	11.282 ± 4.790	7.121 ± 3.552	7.380 ± 1.780	3.901 ± 1.273
MLM	2.201 ± 0.432	1.414 ± 0.325	2.181 ± 0.607	1.260 ± 0.434	11.443 ± 4.365	7.324 ± 3.079	8.099 ± 1.716	4.673 ± 1.366

By quantitative comparison of average RE and SAD values, conclusions consistent with qualitative evaluations were obtained: For all mixing models, VE generally provided superior unmixing accuracy compared to AE, with this advantage being particularly pronounced in rice fields; LMM, Fan, and GBM exhibited relatively poor performance, followed by NM and PPNM, while LQM and MLM demonstrated optimal unmixing results. Most notably, the proposed VE-MLM framework achieved superior performance. It yielded remarkably low RE (sorghum: 1.414 ± 0.325 × 10^−2^; rice: 1.260 ± 0.434 × 10^−2^) and SAD (sorghum: 7.324 × 10^−2^±3.079 × 10^−2^; rice: 4.673 ± 1.366 × 10^−2^), with the lowest Std in the sorghum experiment and a comparable low Std in the rice experiment. This minimal fluctuation confirmed that the VE-MLM framework maintained high stability and reliability even under dynamically changing crop environments.

Based on comprehensive analysis of qualitative and quantitative results, three key conclusions can be drawn: (1) For both sorghum and rice fields, the VE-MLM framework achieved excellent unmixing accuracy, significantly outperforming traditional AE-LMM or VE-LMM (MESMA); (2) For crop canopies, endmember selection significantly impacts the unmixing performance of mixing models and variable endmembers is necessary; (3) Most nonlinear mixing models are better suited for crop growth fields than linear models.

### 3.2 Relationship between foreground abundance data and FAPAR

In SMA, abundance represents the contribution of endmembers to the total spectral signal [32]. Therefore, for crop growth fields, foreground abundance indicates the contribution of the canopy. Visually, the foreground abundance maps based on VE-MLM showed that abundance changes over multiple periods were consistent with the evolution of crops (Fig. S2). Overall, the foreground abundance of soil was essentially zero, and as crops grow, foreground abundance increased, with row-ridge structures transitioning from distinct to blurred.

For sorghum with different sowing dates, late-sown plots with shorter plants generally exhibited growth retardation during the early growth stage (Fig. S2A–C), resulting in significant differences in foreground abundance among different plots. In the late growth stage (Fig. S2D–F), phenological stages of all plots became almost synchronized, with leaves fully expanded in all plots, leading to a marked reduction in foreground abundance differences. For rice with three nitrogen fertilizer levels, nitrogen-deficient plots consistently showed less vigorous growth compared to 1N and 2N plots, correspondingly maintaining lower foreground abundance throughout the period.

Correspondingly, the recollision probability *P* was also adaptively adjusted for different pixels. As illustrated in Fig. S3, the spatial distribution of *P* within individual plots exhibited a distinct pattern consistent with the row-ridge planting structure. Specifically, higher values were observed along the crop ridges, corresponding to dense canopy areas with frequent multiple scattering events, whereas significantly lower values were observed in the inter-row spaces dominated by the background. At the plot level, distinct variations in values were evident across different treatments, particularly distinguishing between indica and japonica rice varieties (Fig. S3G–K). These results demonstrate that *P* can capture the canopy structural heterogeneity.

**Fig. 7:**
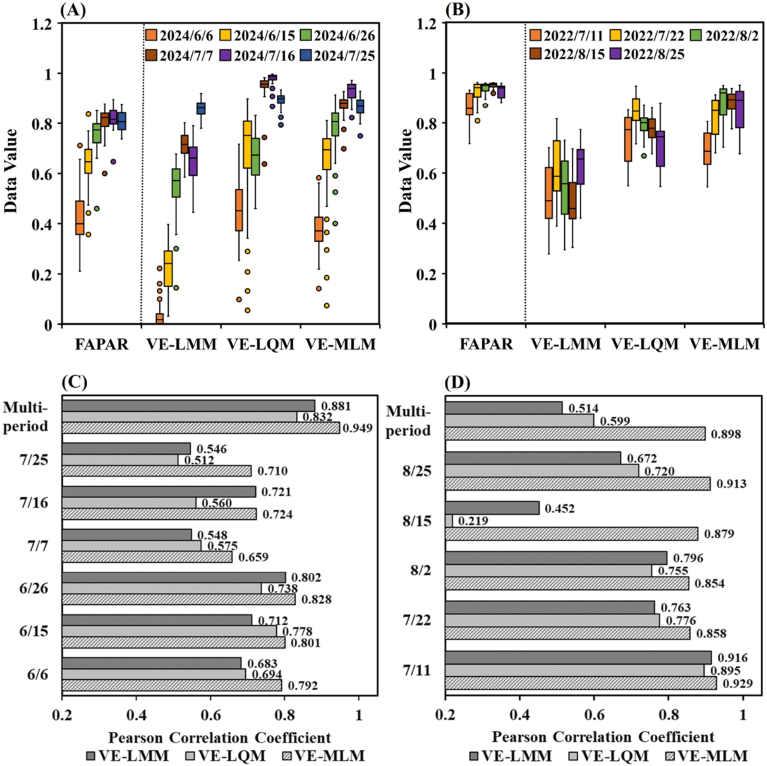
Temporal variations of (A) sorghum and (B) rice FAPAR and foreground abundances from VE-LMM, VE-LQM and VE-MLM, and the correlation coefficient of (C) sorghum and (D) rice between single-period and multi-period FAPAR and foreground abundances.

To further evaluate the effectiveness of VE-MLM, the average foreground abundance of each plot was calculated within each ROI (Section 2.2.1), whose variation trend of time series during crop growth is shown in Fig. 7A (sorghum) and Fig. 7B (rice), with field-measured FAPAR as reference. And Fig. 7C (sorghum) and Fig. 7D (rice) show the Pearson correlation coefficient between foreground abundance and corresponding FAPAR. Additionally, comparisons were made with one linear mixing model, VE-LMM, and one bilinear mixing model, VE-LQM, which exhibited excellent unmixing accuracy. For the VE-LQM, the foreground abundance was defined as the sum of the coefficients of the terms about foreground endmembers.

The FAPAR value directly reflects the light interception capacity of the vegetation canopy. As shown in Fig. 7A–B, during the jointing stage (before 2024/7/7 or 2022/8/15), FAPAR grew rapidly with the continuous increase of crop canopy, and the differences between plots were significant; after heading (2024/7/7 or 2022/8/15), panicle development became dominant with stem and leaf development slowing down, FAPAR tended to saturate, and the differences between fields narrowed. Compared with sorghum, the rice canopy was denser, and the overall FAPAR was higher, approaching 1.

The variation trends of foreground abundance derived from VE-LMM and VE-LQM were irregular, deviating from the variation trends of FAPAR and the actual crop structure changes. The sorghum foreground abundances derived from VE-LMM basically continued to increase, with single-period correlations with FAPAR ranging from 0.546 to 0.802, and multi-period correlation of 0.881; the rice foreground abundances showed fluctuations, with single-period correlations ranging from 0.452 to 0.916, and multi-period correlation of 0.514 (Fig. 7C–D). For VE-LQM, both sorghum and rice foreground abundances generally showed a trend of first increasing and then decreasing. The correlation between sorghum foreground abundances and FAPAR ranged from 0.512 to 0.778, with a multi-period correlation of 0.832; for rice, the correlation ranged from 0.219 to 0.895, with a multi-period correlation of 0.599. The correlations between foreground abundances derived from both methods and FAPAR were better in the early growth stage.

The foreground abundances derived from VE-MLM basically first increased and then saturated, consistent with the FAPAR variation trend. For sorghum, the single-period correlation between foreground abundance and FAPAR remained between 0.659 and 0.828, with relatively lower correlations after heading (2024/7/7), and a multi-period correlation of 0.949; for rice, it remained between 0.851 and 0.929, and a multi-period correlation of 0.898. For both single and multi-period sorghum and rice, the foreground abundances derived from VE-MLM showed a strong positive correlation with FAPAR, significantly outperforming VE-LMM and VE-LQM. This indicates that the VE-MLM framework can more effectively perform unmixing in complex crop growth scenes.

### 3.3 FAPAR estimation using vegetation index and abundance data

**Fig. 8:**
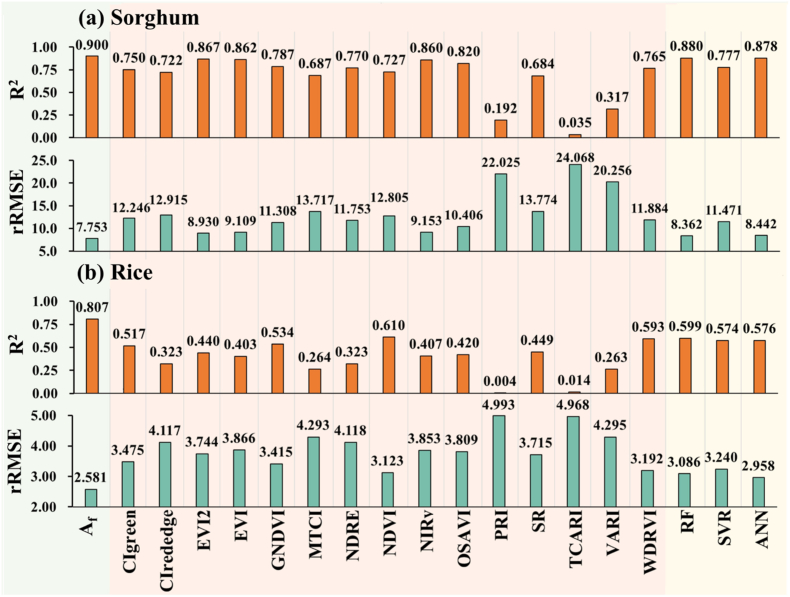
FAPAR estimation accuracy (R^2^ and rRMSE) of the proposed foreground abundance *A*_*f*_, typical VIs, and machine learning models for (A) sorghum and (B) rice at multiple periods.

In this study, simple linear regression was employed to analyze the correlations between multi-period FAPAR and both foreground abundance of VE-MLM and typical VIs (Fig. 8). Additionally, to evaluate the potential of typical VIs for non-linear estimation, three ML models (RF, SVR, ANN) were tested using 5-fold cross-validation. For sorghum, the foreground abundance *A*_*f*_ exhibited an R^2^ of 0.900 and an rRMSE of 7.753% with FAPAR; among the VIs, EVI2 showed the best performance with an R^2^ of 0.867 and an rRMSE of 8.930%, while NDVI yielded an R^2^ of 0.727 and an rRMSE of 12.805%; the RF model achieved the highest accuracy among the ML methods (R^2^ = 0.880, rRMSE = 8.362%). For rice, the *A*_*f*_ achieved an R^2^ of 0.807 and an rRMSE of 2.200% with FAPAR; among the VIs, NDVI demonstrated the optimal performance with an R^2^ of 0.602 and an rRMSE of 3.158% with FAPAR, whereas EVI2 resulted in an R^2^ of 0.434 and an rRMSE of 4.119%; the RF model (R^2^ = 0.599, rRMSE = 3.086%) was the optimal ML and slightly inferior to the simple NDVI regression. Overall, as Fig. 8 shows, the foreground abundance derived from VE-MLM always had the highest potential of estimating FAPAR for both sorghum and rice.

## 4 Discussion

The reflectance received by the sensor is the result of multiple interactions of light within the scene. This study fully considered the dynamic changes of foreground and background in crop growth fields, as well as the complex transmission process of light in the scene, and proposed a VE-MLM framework, based on an adaptive endmember extraction method and multilinear mixing model, which better quantifies the contribution weight of crop canopy to spectral signals, namely foreground abundance *A*_*f*_. Taking sorghum and rice as examples, correlation analysis was conducted between multi-period FAPAR and *A*_*f*_, achieving higher accuracy than traditional VIs and ML models.

### 4.1 The effectiveness of the variable spectral library

The selection of endmembers significantly affects the final unmixing accuracy [31,57]. The “same body with different spectrum” phenomenon is common in farmland, where materials in the scene constantly change dynamically (Fig. 1). Therefore, it is necessary to adaptively establish a spectral library suitable for the current scene. In this study, during each data collection, close-range UAV images were simultaneously acquired to ensure the presence of pure pixels. Aiming at the differences in reflectance characteristics of ground objects, an R-NIR feature space was proposed to quickly screen two classes of pure pixels: one is foreground, which includes green organs such as leaves, stems, and panicles; the other is background, which includes soil or muddy water. Subsequently, K-means and IES algorithms were used to greatly reduce the redundancy of endmember spectra, resulting in the most representative spectral library for the current scene.

Compared with fixed AE, VE generally achieved better unmixing accuracy for most spectral mixing models, with particularly significant improvement for rice (Fig. 6 and Table 1). The endmember spectra with high spectral resolution were measured in different growth stages using an ASD Field Spec 4 spectrometer (Analytical Spectral Devices Inc., Boulder, CO, United States), including foreground organs and background materials. As shown in Fig. S4, it was confirmed that sorghum and rice generally exhibit spectral variability during growth. Sorghum experimental fields were planted with similar glutinous varieties and the soil was relatively more homogeneous, resulting in relatively smaller spectral variability within the scene. Rice experimental fields were planted indica and japonica varieties; meanwhile, muddy water was influenced by water depth and turbidity and had different reflectance. Therefore, regardless of foreground or background, spectral variability was greater in rice growth scenarios. Consequently, the variable spectral library achieved significantly better unmixing results than fixed endmembers for rice. It can be seen that the greater the spectral variability in the application scenario, the more obvious the advantages of the VE spectral library.

### 4.2 Unmixing performance of VE-MLM in crop growth environments

Crops such as sorghum and rice have their organs distributed in a staggered manner in three-dimensional space, forming multi-layer mixed scenes. Different spectral mixing models had different applicability and unmixing results in our crop scenes (Fig. 6 and Table 1). The LMM simplifies the spectral mixing process of complex scenes into a linear weighted combination of endmember spectra. This simplification struggles to cope with additional complexities of light, such as multiple transmissions and scattering, which are prevalent in natural environments. Especially in shadowing and mutual illumination areas (Fig. 6), spectral distortion often occurred in the unmixing results. In other words, the recalculated spectra in these regions deviated significantly from the actual reflectance, which meant certain errors in estimated abundances and ultimately affected the quantitative inversion of FAPAR. Due to the relatively sparse canopy of sorghum, with larger and looser panicles and leaves, heterogeneity in both horizontal and vertical directions is more pronounced. The LMM's neglect of multiple scattering made it difficult to accurately describe the optical transmission in the complex scene, thus, the limitations of LMM were particularly prominent in the sorghum experiment.

The BMMs introduce quadratic terms to describe the secondary interaction of photons, with different definitions of quadratic terms and constraints leading to different compatibility with practical scenarios [35]. As shown in Table 1, the Fan model's quadratic term magnitude depends entirely on linear terms, resulting in the worst unmixing performance; the GBM and PPNM incorporate quadratic term scaling parameters, but linear term coefficients still restrict quadratic term magnitudes, and scaling parameters tend to be negative, causing nonlinear terms to become negative; the NM and LQM assume that bilinear terms can be treated as extra endmembers, that is to say, the probability of secondary interactions in the canopy is relatively independent of the probability of linear reflections, achieving excellent unmixing accuracy. The MLM further describes complex light propagation processes in the three-dimensional scenes, considering multiple transmission and scattering, and achieved relatively optimal unmixing accuracy and stability in both sorghum and rice experiments.

**Fig. 9:**
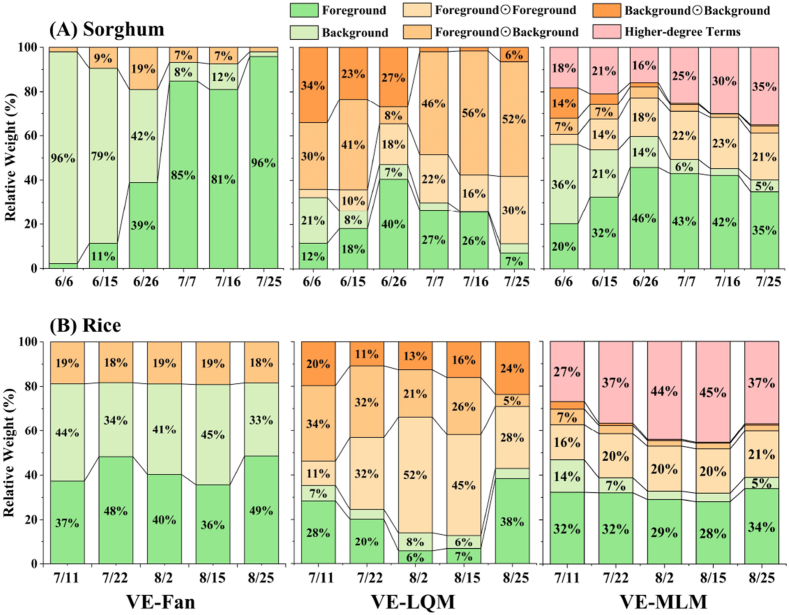
Relative weight of each item of VE-Fan, VE-LQM and VE-MLM for (A) sorghum and (B) rice.

To further analyze the reasons for different unmixing results among these models, Fig. 8 shows the temporal variation of each coefficient for VE-Fan, VE-LQM, and VE-MLM in sorghum and rice experiments, with percentages representing the average relative weight of each item to the overall spectrum during that period (terms below 5% are not labeled in the figure). For VE-MLM, the relative weight of each item was calculated through the obtained abundances and *P* by Eq. (5), and the contribution of higher-degree terms was the approximate sum of third-order and higher terms. As shown in Fig. 9, the VE-Fan's quadratic term relative weight was generally below 20%, while VE-LQM's quadratic terms contributed about 50%-90%. The VE-MLM's bilinear terms contributed about 20%, and higher-order interactions contributed 18%-45%. It can be seen that for any growth period, nonlinear interactions of light in our crop growth scenes were significant and cannot be ignored. When a spectral mixing model provides sufficient degrees of freedom for nonlinear terms, its applicability to current scenes may be higher.

However, the unmixing accuracy does not directly represent the validity of the results [58]. In Fig. 7, the foreground abundance derived from VE-LQM showed irregular temporal variations. The material mixing method and physical mechanism of LQM are not well-suited for the growth scenarios of sorghum and rice. Based on Fig. 9, we found that the contribution of the linear term in VE-LQM was far smaller than that of the quadratic term. Thus, it was speculated that when the contribution of the single reflection did not constrain the probability of multiple interactions occurring, it may obtain solutions that do not conform to physical meaning. In the MLM, the probability of linear or nonlinear interactions depends entirely on the abundances and light recollision probability *P*, which ensures that the contribution of each term gradually decreases. Both the abundances and coefficients of VE-MLM exhibited regular temporal changes, consistent with the actual crop development.

In summary, the LMM or BMMs simplify the interaction process between light and materials, and have certain limitations in multi-layered crop growth scenarios. The VE-MLM model can better describe the optical complex transmission in the canopy and has stronger applicability to the specific scenarios.

### 4.3 Potentials of *A*_*f*_ in FAPAR estimation

**Fig. 10:**
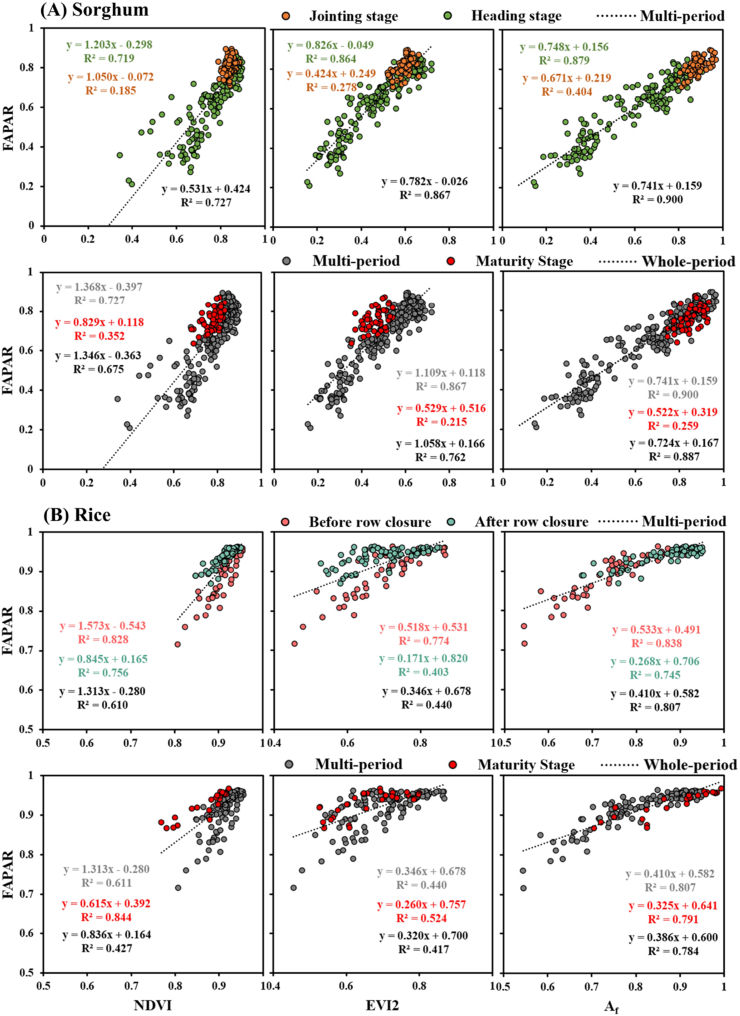
The linear regression results of NDVI, EVI2 and the proposed foreground abundance *A*_*f*_ versus FAPAR for (A) sorghum and (B) rice at different periods.

A simple linear regression was performed between the foreground abundance *A*_*f*_ derived from VE-MLM and multi-period FAPAR, achieving higher accuracy than typical VIs. To provide a more comprehensive comparison, we also evaluated three non-linear ML models using these VIs as inputs with 5-fold cross-validation, further demonstrating the advantages of VE-MLM. VIs did not yield consistent results across the two crops, indicating that the tested VI was not fully suitable for FAPAR inversion in both sorghum and rice. Fig. 10 illustrates the linear relationships between FAPAR and EVI2, NDVI, and *A*_*f*_ of sorghum and rice. EVI2 and NDVI were the most accurate VIs for estimating FAPAR of the two crops, respectively.

In the sorghum experiment, VIs exhibited significant saturation after heading. Sorghum canopies are relatively loose, with rapid volume expansion during the jointing stage, leading to a strong positive linear correlation between VIs and FAPAR (EVI2: R^2^ = 0.864). After heading, stem and leaf development delays, and green, spindle-shaped inflorescences form and continuously expand at the top, resulting in more complex canopy structures and increased multiple scattering. However, VIs became insensitive to FAPAR changes and entered a saturation state after heading, causing the R^2^ of EVI2 to drop to 0.278. The final multi-period estimation accuracy was 0.867.

In the rice experiment, VIs showed distinct segmentation before and after row closure, particularly NDVI. Before closure, more background was exposed, and the R^2^ of NDVI and FAPAR was 0.828. After closure, the canopy became dense and compact, significantly reducing background contributions to the spectrum, with an R^2^ of 0.756. Rice grows in a water-soil mixture background, leading to high spectral variability [53]. VIs were affected differently by background interference in the two stages, resulting in significant differences in the slopes of the linear relationships with FAPAR and reducing the multi-period estimation accuracy to R^2^ = 0.610.

Compared to VIs, *A*_*f*_ demonstrated strong linear correlations with multi-period FAPAR for both sorghum (R^2^ = 0.900) and rice (R^2^ = 0.807). The *A*_*f*_ from VE-MLM represents the probability of photon collisions with foreground materials [32], while FAPAR positively correlates with the canopy photon average interception probability [11], implying inherent physical consistency between *A*_*f*_ and FAPAR. Furthermore, *A*_*f*_ maintained more consistent linear relationships across sorghum heading and rice row closure stages, indicating VE-MLM was less affected by crop dynamic growth and environmental interference. Thus, it was validated that the impact of spectral mixture for VI-based FAPAR estimation methods cannot be ignored, and the VE-MLM framework had robust responsiveness to the complex crop environments.

The maturity stage presents a unique challenge for remote sensing estimation due to the changes in the canopy composition. During the maturity stage, sorghum panicles turn red and rice panicles turn yellow, leading to a more complex foreground composition. As shown in Fig. 1, the red band reflectance of red/yellow panicle endmembers was higher than that of green organs, while the reflectance in NIR was lower. This caused fluctuations in VIs, especially composed of R and NIR, such as NDVI and EVI2. Specifically, for sorghum, the R^2^ of EVI2 decreased from 0.867 to 0.762, and for rice, the R^2^ of NDVI decreased from 0.611 to 0.427. The extensibility of VE-MLM was also tested. Pure panicle pixels were extracted from the R-NIR space, and together with green organs and the background, they form a spectral library containing three types of endmembers. The sum of green organ abundance and panicle abundance derived from the VE-MLM was defined as *A*_*f*_. As illustrated in Fig. 10, compared with VIs, *A*_*f*_ exhibited a more consistent linear relationship both before and after panicle discoloration, whose R^2^ changed from 0.900 to 0.887 in the sorghum experiment and from 0.807 to 0.784 in the rice experiment. This further verified that the VE-MLM had a good response to dynamic changes of objects in scenes and possessed the ability to estimate crop FAPAR throughout the entire growth period.

### 4.4 Scalability and potential for satellite applications

Although VE-MLM demonstrates high accuracy at the plot scale, applying it to large-scale fields poses challenges regarding data acquisition efficiency and computational cost. Collecting full-coverage close-range UAV imagery for extensive areas is constrained by flight efficiency and data volume. To enhance operational feasibility in larger agricultural scenarios, we suggest adopting a representative sampling strategy: establishing the spectral library using high-resolution data from small, representative sub-areas, and subsequently applying it to unmix the full-coverage imagery of the entire field. This approach is expected to significantly improve operational efficiency without compromising the unmixing framework's physical mechanism. In addition to data acquisition, future research will focus on optimizing the endmember combination iterative selection algorithm to further reduce runtime and facilitate the rapid processing of large-scale datasets.

Furthermore, extending the framework to satellite platforms is constrained by the sensors' spatial and spectral resolutions. Spatial resolution restricts the direct building of the variable spectral library. The scarcity of pure pixels in coarse-resolution satellite imagery hinders image-based endmember extraction. To overcome this, on the one hand, future research will focus on satellite-UAV/ground synergy. The variable spectral library can be established using synchronous high-resolution UAV data or ground measurements. In the absence of synchronous data, historical spectral libraries can serve as prior knowledge. On the other hand, advanced automated endmember generation algorithms can be applied to derive endmember bundles from mixed pixels. Future work will further explore the significant potential of these algorithms in complex agricultural environments, thereby overcoming the reliance on pure pixel identification for satellite applications. Besides, the spectral resolution of satellite sensors influences the solution of MLM. Mathematically, the nonlinear spectral mixing model requires the band number to exceed or equal *p+1* (where *p* is the number of endmember classes and the additional ‘1’ accounts for the unknown recollision probability *P*), which remains a challenge for sensors with limited spectral bands.

Overall, aiming at spectral variability and complex spatial structures in crop growth scenarios, this study proposed the VE-MLM framework, which calculated the canopy abundance *A*_*f*_ from pixel reflectance. This framework accurately quantified the contribution of the dynamically changing crop canopy to spectral signals and better described the scattering and transmission processes in crop scenes at multiple periods. Correlation analysis between *A*_*f*_ and multi-period FAPAR of sorghum and rice demonstrated a strong linear relationship. Compared with typical VIs and ML models, the VE-MLM framework can flexibly adapt to the dynamic changes and complexity of the crop scenes and exhibit more robust responsiveness.

## 5 Conclusion

This study proposed an adaptive spectral unmixing framework, VE-MLM, to address the challenges of spectral variability and scene complexity in crop FAPAR estimation. By selecting the optimal combination based on the variable endmember library and the multilinear mixing model, the framework effectively derives the foreground abundance *A*_*f*_. Through an ablation study on the key modules of VE-MLM, we verified the necessity and effectiveness of VE spectral library and MLM, which significantly outperformed fixed-endmember and linear/bilinear models in crop scenarios. Furthermore, the physical significance of *A*_*f*_ was discussed and analyzed, revealing a significant linear correlation with FAPAR of sorghum and rice, and it was concluded that *A*_*f*_ can effectively quantify the canopy contribution of light propagation to the sensor-received signals. Comparisons with typical VIs demonstrated that VE-MLM offers superior robustness against dynamic growth changes and background interference, achieving excellent results in multi-period FAPAR estimation for multi-variety sorghum (R^2^ = 0.900) and rice (R^2^ = 0.807). These results outperform the optimal VIs (Sorghum-EVI2: R^2^ = 0.867; Rice-NDVI: R^2^ = 0.610) and the optimal ML model (Sorghum-RF: R^2^ = 0.880; Rice-RF: R^2^ = 0.599).

Despite these promising results, certain limitations remain. The current framework relies on identifying pure pixels from high-resolution UAV imagery for endmember extraction, which restricts its direct application to coarse-resolution satellite data where pure pixels are scarce. Additionally, the pixel-by-pixel iterative unmixing process incurs a higher computational cost. Future research will focus on: (1) introducing endmember generation algorithms to adapt the framework to satellite data; (2) optimizing the endmember selection algorithm to improve computational efficiency; and (3) validating the model's robustness on a wider variety of crops and under more diverse environmental conditions.

## CRediT authorship contribution statement

Ningge Yuan: Writing - original draft, Investigation, Conceptualization, Methodology, Software, Visualization. Yadong Liu: Conceptualization, Investigation, Data Curation. Chaoran Zhang: Investigation, Data curation. Yuanjin Li: Investigation, Data Curation. Longfei Ma: Investigation, Data Curation. Yi Peng: Conceptualization, Supervision, Funding acquisition. Xianting Wu: Resources; Renshan Zhu: Resources; Yan Gong: Writing - review & editing, Methodology, Project administration, Funding acquisition. Shenghui Fang: Supervision, Resources, Funding acquisition.

## Funding

This work was supported by Kweichow Moutai Co., Ltd. (university-enterprise cooperation project: MTGF2023047, MTGF2023048); the Department of Natural Resources of Hubei Province (provincial-ministry collaborative pilot project: 2023ZRBSHZ025); the State Administration of Science, Technology and Industry for National Defense (advanced research project: Remote sensing inversion of key land surface elements and development of quantitative products); and the Basic Surveying and Mapping Information Center of Anhui Province (provincial-level natural resources science and technology project: 2023-K-2).

## Declaration of competing interest

The authors declare that they have no known competing financial interests or personal relationships that could have appeared to influence the work reported in this paper.

## Data Availability

The data collected and/or analyzed during this study can be obtained from the corresponding author upon reasonable request.
