# Supplementary material for: VE-MLM: A variable endmember-based multilinear mixing framework for crop FAPAR estimation using UAV multispectral imagery
Source: Plant Phenomics. 2026 Apr 4;8(2):100202. doi: 10.1016/j.plaphe.2026.100202 (PMC13316481; doi:10.1016/j.plaphe.2026.100202)
Supplement: Multimedia component 1 [file mmc1.docx]

Supplementary Materials


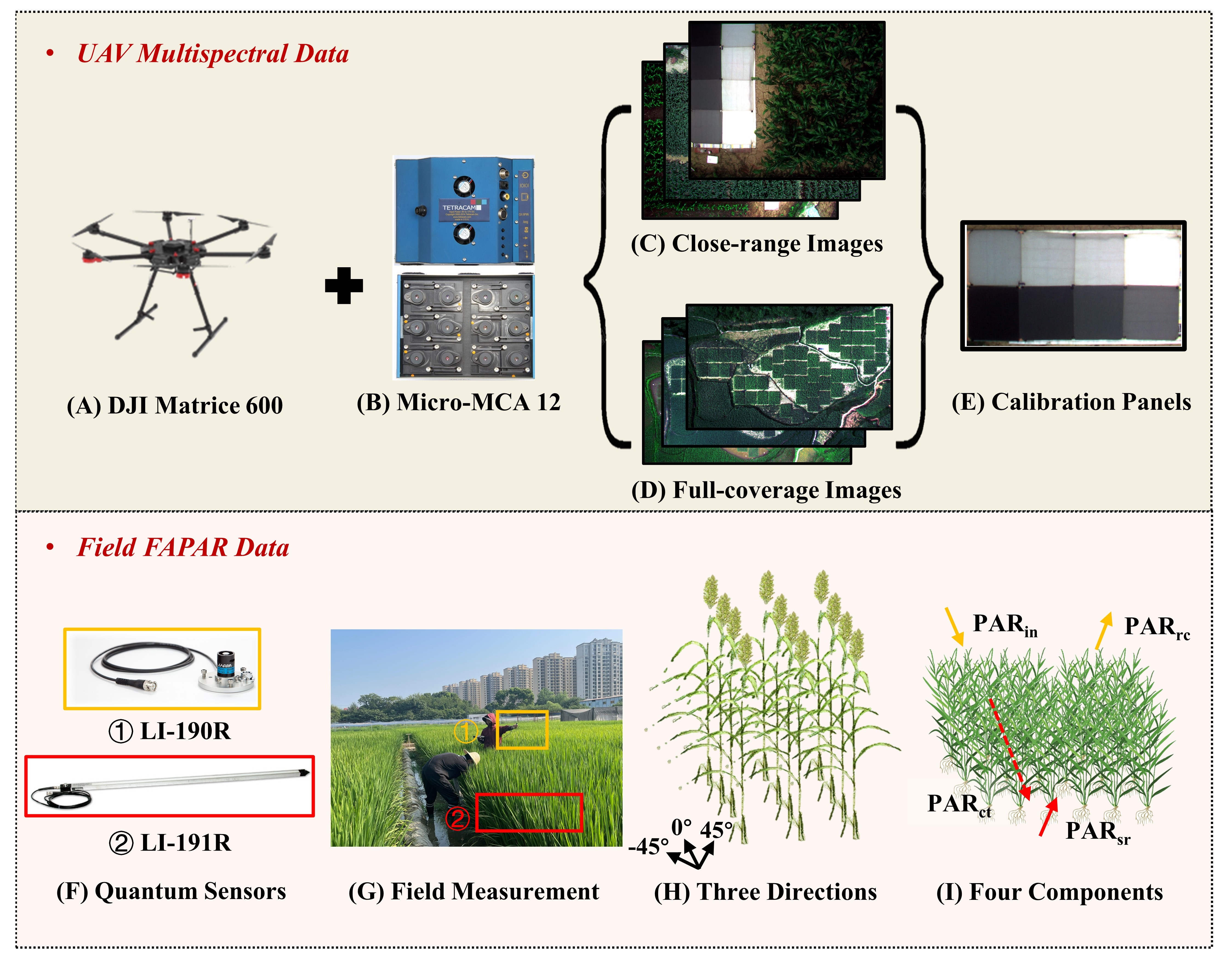


Fig.S1 Measurement of UAV multispectral data and field FAPAR data.


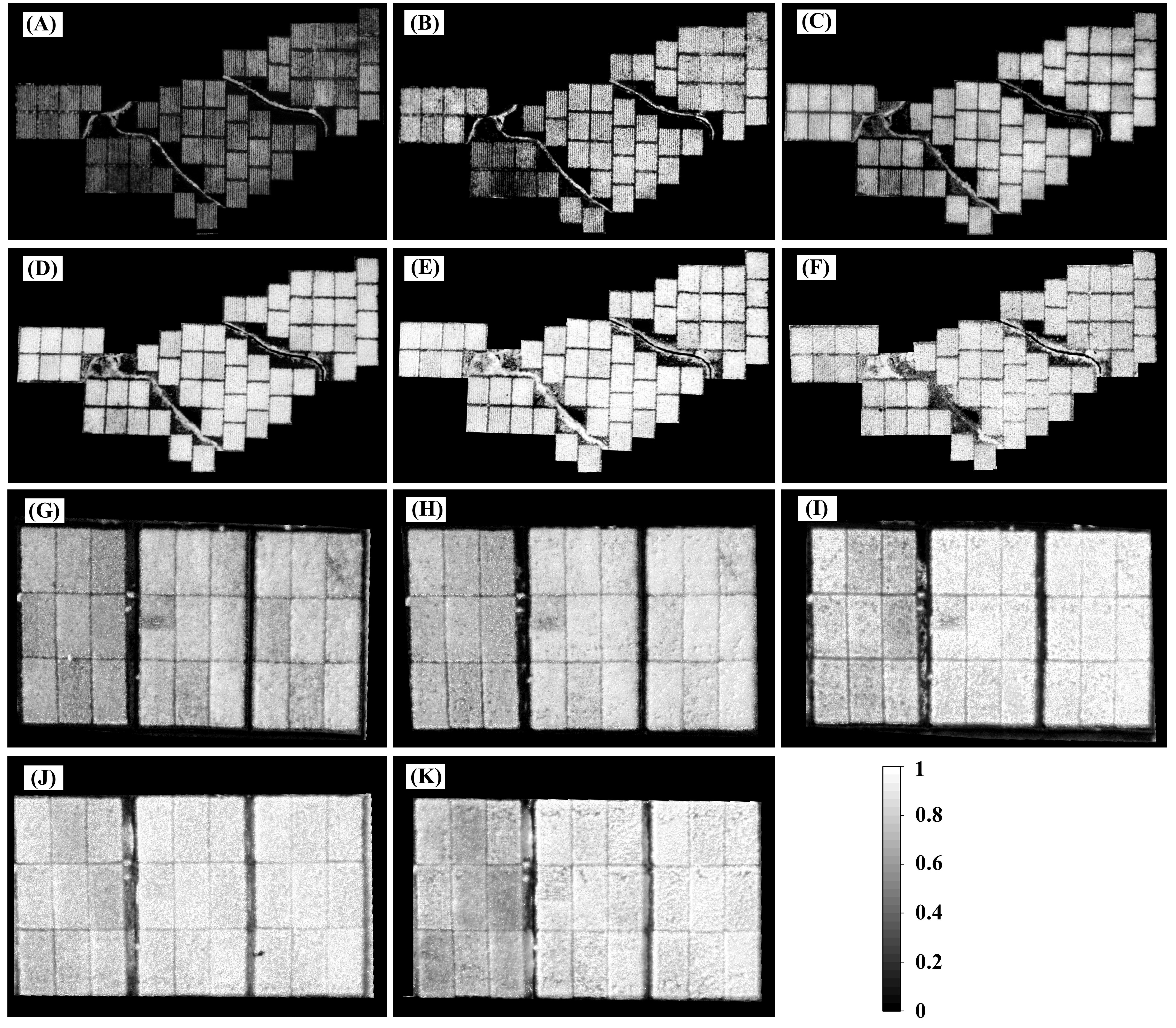


Fig.S2 Foreground abundance maps of sorghum on (A) 2024/6/6, (B) 2024/6/15, (C) 2024/6/26, (D) 2024/7/7, (E) 2024/7/16, (F) 2024/7/25 and rice on (G) 2022/7/11, (H) 2022/7/22, (I) 2022/8/2, (J) 2022/8/15, (K) 2022/8/22.


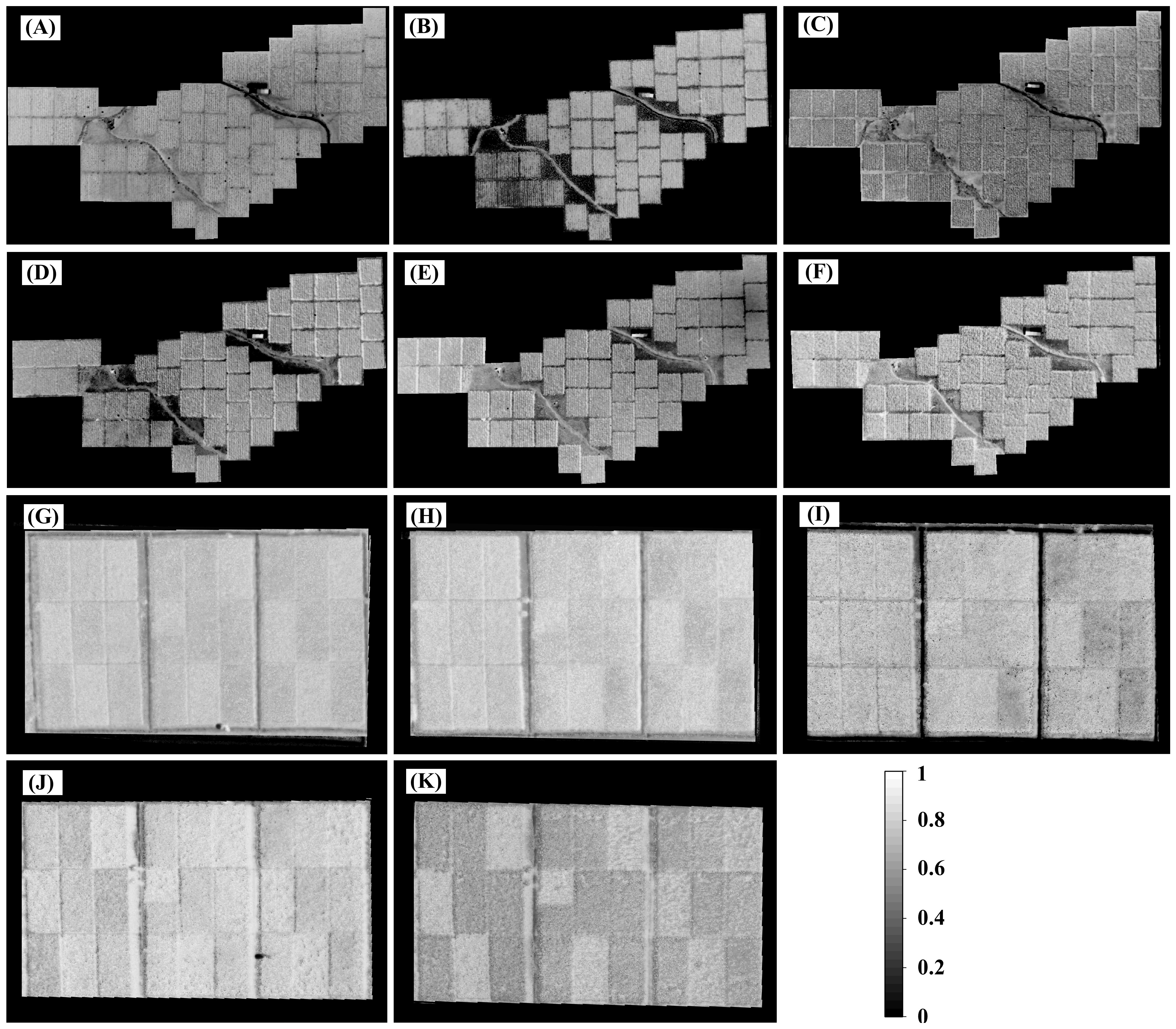


Fig.S3 Recollision probability *P* maps of sorghum on (A) 2024/6/6, (B) 2024/6/15, (C) 2024/6/26, (D) 2024/7/7, (E) 2024/7/16, (F) 2024/7/25 and rice on (G) 2022/7/11, (H) 2022/7/22, (I) 2022/8/2, (J) 2022/8/15, (K) 2022/8/22.


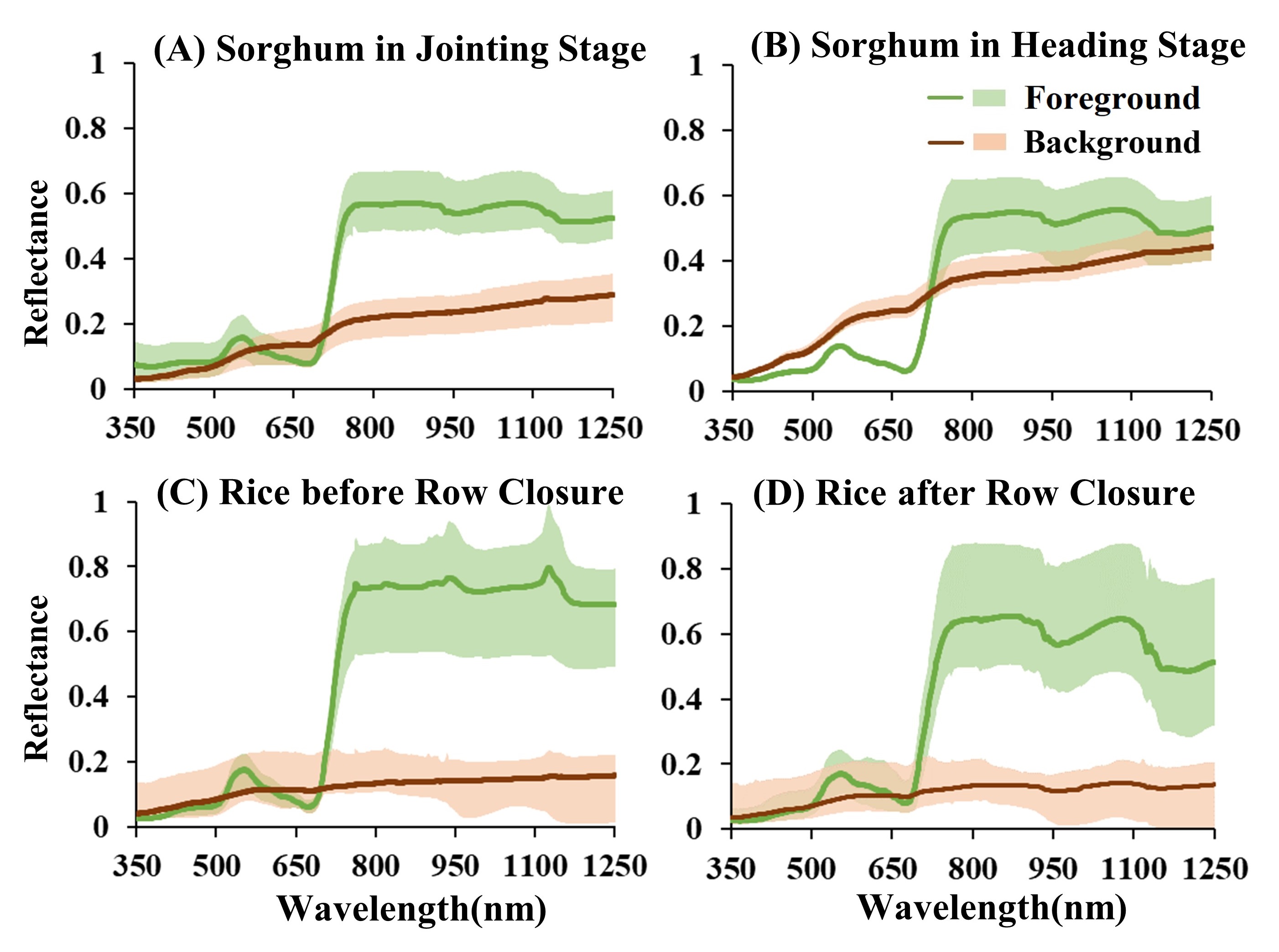


Fig.S4 The measured ground hyperspectral reflectance of endmembers of sorghum at (A) jointing stage and (B) heading stage, and rice (C) before and (D) after row closure. The colored bars are the spectral variation ranges. Note: The in situ spectra were measured from the crop fields using an ASD Field Spec 4 spectrometer (Analytical Spectral Devices Inc., Boulder, CO, United States)

Table S1. Vegetation indices tested in this study.

| **Vegetations Indices** | **Formula** | **Reference** |
| --- | --- | --- |
| Green Chlorophyll Index (CI_green_) | $\rho_{800}/\rho_{550}-1$ | [1] |
| Red-edge Chlorophyll Index (CI_red edge_) | $\rho_{800}/\rho_{720}-1$ | [1] |
| Two-band Enhanced Vegetation Index (EVI2) | $2.5\left(\rho_{800}-\rho_{670}\right)/\left(\rho_{800}+2.4\rho_{670}+1\right)$ | [2] |
| Enhanced Vegetation Index (EVI) | $2.5\left(\rho_{800}-\rho_{670}\right)/\left(\rho_{800}+6\rho_{670}-7.5\rho_{490}+1\right)$ | [3] |
| Green Normalized Difference Vegetation Index (GNDVI) | $\left(\rho_{800}-\rho_{550}\right)/\left(\rho_{800}+\rho_{550}\right)$ | [4] |
| MERIS Terrestrial ChlorophyⅠⅠ Index (MTCI) | $\left(\rho_{800}-\rho_{720}\right)/\left(\rho_{720}-\rho_{670}\right)$ | [5] |
| Normalized Difference Red Edge Vegetation Index (NDRE) | $\left(\rho_{800}-\rho_{720}\right)/\left(\rho_{800}+\rho_{720}\right)$ | [6] |
| Normalized Difference Vegetation Index (NDVI) | $\left(\rho_{800}-\rho_{670}\right)/\left(\rho_{800}+\rho_{670}\right)$ | [7] |
| Near-Infrared Reflectance of Vegetation (NIRv) | $\rho_{800}\times\left(\rho_{800}-\rho_{670}\right)/\left(\rho_{800}+\rho_{670}\right)$ | [8] |
| Optimized Soil Adjusted Vegetation Index (OSAVI) | $\left(1+0.16\right)\left(\rho_{800}-\rho_{720}\right)/\left(\rho_{800}+\rho_{720}+0.16\right)$ | [9] |
| Photochemical Reflectance Index (PRI) | $\left(\rho_{520}-\rho_{570}\right)/\left(\rho_{520}+\rho_{570}\right)$ | [10] |
| Simple Ratio Index (SR) | $\rho_{800}/\rho_{670}$ | [11] |
| Transformed Chlorophyll Absorption Ratio Index (TCARI) | $3\times\left(\rho_{800}-\rho_{720}\right)/\left(\rho_{800}+\rho_{720}+0.16\right)$ | [12] |
| Visible Atmospherically Resistant Index (VARI) | $\left(\rho_{550}-\rho_{670}\right)/\left(\rho_{550}+\rho_{670}-\rho_{490}\right)$ | [13] |
| Wide Dynamic Range Vegetation Index (WDRVI) | $\left(\alpha\times\rho_{800}-\rho_{670}\right)/\left(\alpha\times\rho_{800}+\rho_{670}\right)\alpha=0.2$ | [14] |

Table S2. Parameter settings for the machine learning models

| **Machine Learning Model** | **Parameters** |
| --- | --- |
| Random forest | n_estimators=100  min_samples_leaf=5  bootstrap=True (default)  criterion=MSE (default)  random_state=42 |
| Support vector regression | kernel='gaussian' (RBF)  gamma='auto' (Heuristic)  C=1.0 (default)  epsilon=estimated (default)  preprocessing = Min-Max Normalization [0,1] |
| Artificial neural networks | structure=[15, 10, 1]  hidden_layer_size=10  algorithm='Levenberg-Marquardt' activation='tansig' (hidden);'purelin' (output)  max_epochs=1000  preprocessing = Min-Max Normalization [0,1] |

Table S3. Classical mixing models

| **Model** | **Formula** | **Algorithm** | **Reference** |
| --- | --- | --- | --- |
| LMM | $x=\sum_{i=1}^{p} a_{i}e_{i}+\xi,\sum_{i=1}^{p} a_{i}=1,a_{i}\geq0$ | FCLS | [15] |
| Fan | $x=\sum_{i=1}^{p} a_{i}e_{i}+\sum_{i=1}^{p-1} \sum_{j=i+1}^{p} a_{i}a_{j}\left(e_{i}\odot e_{j}\right)+\xi,\sum_{i=1}^{p} a_{i}=1,a_{i}\geq0$ | FCLS | [16] |
| GBM | $x=\sum_{i=1}^{p} a_{i}e_{i}+\sum_{i=1}^{p-1} \sum_{j=i+1}^{p} \gamma_{i,j}a_{i}a_{j}\left(e_{i}\odot e_{j}\right)+\xi,\sum_{i=1}^{p} a_{i}=1,a_{i}\geq0,0\leq\gamma_{i,j}\leq0$ | Gradient | [17] |
| PPNM | $x=\sum_{i=1}^{p} a_{i}e_{i}+\gamma\sum_{i=1}^{p} \sum_{j=i}^{p} a_{i}a_{j}\left(e_{i}\odot e_{j}\right)+\xi,\sum_{i=1}^{M} a_{i}=1,a_{i}\geq0$ | Gradient | [18] |
| NM | $x=\sum_{i=1}^{p} a_{i}e_{i}+\sum_{i=1}^{p-1} \sum_{j=i+1}^{p} \gamma_{i,j}\left(e_{i}\odot e_{j}\right)+\xi,\sum_{i=1}^{p} a_{i}+\sum_{i=1}^{p-1} \sum_{j=i+1}^{p} \gamma_{i,j}=1,a_{i}\geq0,\gamma_{i,j}\geq0$ | FCLS | [19] |
| LQM | $x=\sum_{i=1}^{p} a_{i}e_{i}+\sum_{i=1}^{p} \sum_{j=i}^{p} \gamma_{i,j}\left(e_{i}\odot e_{j}\right)+\xi,\sum_{i=1}^{p} a_{i}+\sum_{i=1}^{p} \sum_{j=i}^{p} \gamma_{i,j}=1,a_{i}\geq0,\gamma_{i}\geq0$ | FCLS | [20] |

Reference

[1] Gitelson AA, Viña A, Ciganda V, Rundquist DC, Arkebauer TJ. Remote estimation of canopy chlorophyll content in crops -: art. no. L08403. Geophys Res Lett. 2005; 32(8). https://doi.org/10.1029/2005gl022688

[2] Jiang ZY, Huete AR, Didan K, Miura T. Development of a two-band enhanced vegetation index without a blue band. Remote Sens Environ. 2008; 112(10):3833-45. https://doi.org/10.1016/ j.rse. 2008.06.006

[3] Liu HQ, Huete A. A feedback based modification of the NDVI to minimize canopy background and atmospheric noise (Vol 33, Pg 457, 1995). IEEE Trans Geosci Remote Sens. 1995; 33(3):814-.

[4] Gitelson AA, Kaufman YJ, Merzlyak MN. Use of a green channel in remote sensing of global vegetation from EOS-MODIS. Remote Sens Environ. 1996; 58(3):289-98.https://doi.org/ 10.1016/S0034-4257(96)00072-7

[5] Dash J, Curran PJ. The MERIS terrestrial chlorophyll index. Int J Remote Sens. 2004; 25(23):5403-13.https://doi.org/10.1080/0143116042000274015

[6] Fitzgerald G, Rodriguez D, O'Leary G. Measuring and predicting canopy nitrogen nutrition in wheat using a spectral index-The canopy chlorophyll content index (CCCI). Field Crops Res. 2010; 116(3):318-24.https://doi.org/10.1016/j.fcr.2010.01.010

[7] Huete AR, Jackson RD, Post DF. Spectral response of a plant canopy with different soil backgrounds. Remote Sens Environ. 1985; 17(1):37-53.https://doi.org/10.1016/0034-4257(85) 90111-7

[8] Badgley G, Field CB, Berry JA. Canopy near-infrared reflectance and terrestrial photosynthesis. Sci Adv. 2017; 3(3). https://doi.org/10.1126/sciadv.1602244

[9] Steven MD. The sensitivity of the OSAVI vegetation index to observational parameters. Remote Sens Environ. 1998; 63(1):49-60. https://doi.org/10.1016/S0034-4257(97)00114-4

[10] Gamon JA, Penuelas J, Field CB. A narrow-waveband spectral index that tracks diurnal changes in photosynthetic efficiency. Remote Sens Environ. 1992; 41(1):35-44. https://doi.org/ 10.1016/0034-4257(92)90059-S

[11] Chen JM. Evaluation of vegetation indices and a modified simple ratio for boreal applications. Can J Remote Sens. 1996; 22(3):229-42. https://doi.org/10.1080/07038992.1996. 10855178

[12] Wu CY, Niu Z, Tang Q, Huang WJ. Estimating chlorophyll content from hyperspectral vegetation indices: Modeling and validation. Agric For Meteorol. 2008; 148(8-9):1230-41. https://doi.org/10.1016/j.agrformet.2008.03.005

[13] Gitelson AA, Kaufman YJ, Stark R, Rundquist D. Novel algorithms for remote estimation of vegetation fraction. Remote Sens Environ. 2002; 80(1):76-87. https://doi.org/10.1016/S0034-4257(01)00289-9

[14] Gitelson AA. Wide dynamic range vegetation index for remote quantification of biophysical characteristics of vegetation. J Plant Physiol. 2004; 161(2):165-73. https://doi.org/10.1078/0176-1617-01176

[15] Heinz DC, Chang CI. Fully constrained least squares linear spectral mixture analysis method for material quantification in hyperspectral imagery. IEEE Trans Geosci Remote Sens. 2001; 39(3):529-45.https://doi.org/ 10.1109/36.911111

[16] Fan WY, Hu BX, Miller J, Li MZ. Comparative study between a new nonlinear model and common linear model for analysing laboratory simulated-forest hyperspectral data. Int J Remote Sens. 2009; 30(11):2951-62.https://doi.org/10.1080/01431160802558659

[17] Halimi A, Altmann Y, Dobigeon N, Tourneret JY. Nonlinear unmixing of hyperspectral images using a generalized bilinear model. IEEE Trans Geosci Remote Sens. 2011; 49(11):4153-62. https://doi.org/10.1109/Tgrs.2010.2098414

[18] Altmann Y, Halimi A, Dobigeon N, Tourneret JY. Supervised nonlinear spectral unmixing using a polynomial post nonlinear model for hyperspectral imagery. Int Conf Acoust Spee. https://doi.org/10.1109/TIP.2012.2187668

[19] José MPN, José MB-D: Nonlinear mixture model for hyperspectral unmixing. ProcSPIE, 2009. p. 74770I. https://doi.org/10.1117/12.830492

[20] Meganem I, Déliot P, Briottet X, Deville Y, Hosseini S. Linear–quadratic mixing model for reflectances in urban environments. IEEE Trans Geosci Remote Sens. 2014; 52(1):544-58. https://doi.org/10.1109/TGRS.2013.2242475
